# Semiconductor Electronic Label-Free Assay for Predictive Toxicology

**DOI:** 10.1038/srep24982

**Published:** 2016-04-27

**Authors:** Yufei Mao, Kyeong-Sik Shin, Xiang Wang, Zhaoxia Ji, Huan Meng, Chi On Chui

**Affiliations:** 1 Department of Electrical Engineering, University of California, Los Angeles, CA 90095, USA; 2 Division of NanoMedicine, Department of Medicine, University of California, Los Angeles, CA 90095, USA; 3 Center for Environmental Implications of Nanotechnology, University of California, Los Angeles, CA 90095, USA; 4 Department of Bioengineering, University of California, Los Angeles, CA 90095, USA; 5 California NanoSystems Institute, University of California, Los Angeles, CA 90095, USA

## Abstract

While animal experimentations have spearheaded numerous breakthroughs in biomedicine, they also have spawned many logistical concerns in providing toxicity screening for copious new materials. Their prioritization is premised on performing cellular-level screening *in vitro*. Among the screening assays, secretomic assay with high sensitivity, analytical throughput, and simplicity is of prime importance. Here, we build on the over 3-decade-long progress on transistor biosensing and develop the holistic assay platform and procedure called semiconductor electronic label-free assay (SELFA). We demonstrate that SELFA, which incorporates an amplifying nanowire field-effect transistor biosensor, is able to offer superior sensitivity, similar selectivity, and shorter turnaround time compared to standard enzyme-linked immunosorbent assay (ELISA). We deploy SELFA secretomics to predict the inflammatory potential of eleven engineered nanomaterials *in vitro*, and validate the results with confocal microscopy *in vitro* and confirmatory animal experiment *in vivo*. This work provides a foundation for high-sensitivity label-free assay utility in predictive toxicology.

With the advent of high-throughput analytical approaches, biomedical scientists have begun to deal with big data sets which rely on innovating and utilizing discovery tools capable of generating rich data in a rapid manner. The same trend is also evident in toxicology which has been facing the challenge of assessing safety profiles of over 90,000 existing and emerging chemicals or materials to humans. Nevertheless, the reality is that such needs are far from satisfied due to both cost and throughput constraints. While assessments have been performed in an observational-based descriptive toxicological fashion using animals, same results can, in our opinion, be obtained more effectively in a predictive manner with target-specific and mechanism-based biological observations^1^,^2^,^3^,^4^,^5^,^6^. This is in agreement with the vision of the National Academy of Sciences (NAS), which advocates a paradigm shift in the approach to toxicological testing in the 21^st^ century, including the development of robust scientific platforms for screening a large number of toxicants at the cellular level^1^. Central to the implementation of predictive toxicology is the move from predominantly animal screening to *in vitro* studies where appropriate^2^,^7^,^8^,^9^. In one embodiment, the data of secretomic assays are utilized to rank material toxicity *in vitro* and predict their toxicity *in vivo*, offering great value in planning and prioritizing the expensive and time-consuming animal experiments for validation purpose.

Most biomolecular assays, such as the enzyme-linked immunosorbent assay (ELISA), are labeling-based, thus have limited analytical throughput and/or sensitivity, involve tedious experimental procedures, and also necessitate costly infrastructure, reagents, and trained personnel. Highly specific biomolecular quantitation can instead be performed with great simplicity via a label-free route, such as the optics-free and enzyme-free field-effect transistor (FET) immunoassay. A FET without a gate electrode designed to detect immunological antigen-antibody binding reactions without labels was patented in 1980^10^ and first unambiguously demonstrated in 1989^11^. The same underlying FET sensor channel has then shrunk to the nanoscale, lain down on an insulator, and evolved to become non-planar^12^. Numerous FET biosensors with nanowire-, nanoribbon-, or nanotube-on-insulator channels have thus emerged^13^,^14^,^15^,^16^,^17^ and demonstrated outstanding lower limits of detection (LLOD) when combined with proper electrical biasing^18^. Nevertheless, their output-signal levels at LLOD are inherently very low, *i.e.* tens to hundreds of picoamps of drain current change as summarized in Supplementary Data Fig. 1, and are therefore impractical for use outside of the research lab. In addition, most FET immunoassays have not been validated against current analytical standards such as the gold standard ELISA, and have not been deployed in quantitating real biological samples in the scenario of interest. As a result, label-free FET immunoassays have not been meaningfully adopted and impacted the biomedical and pharmaceutical industry even after over 3 decades of intense research.

## Results

### SELFA development and analytical validations

The low output signal level of an FET sensor can be readily boosted in the electrical domain, yet such amplification should occur as close as possible to the sensor in order to minimize the noise induced by parasitic components^19^. One feasible concept is an integrated nanowire FET sensor-amplifier that can locally amplify the transduced sensing current^20^,^21^. In this work, we developed such sensor-amplifier concept into a biomolecular assay platform for the first time, which we call Semiconductor Electronic Label-Free Assay (SELFA). In particular, we explored the viability of the on-the-sensing-spot amplification in immunodetection, developed a calibration strategy, and performed analytical validation of the holistic immunoassay.

**Figure 1:**
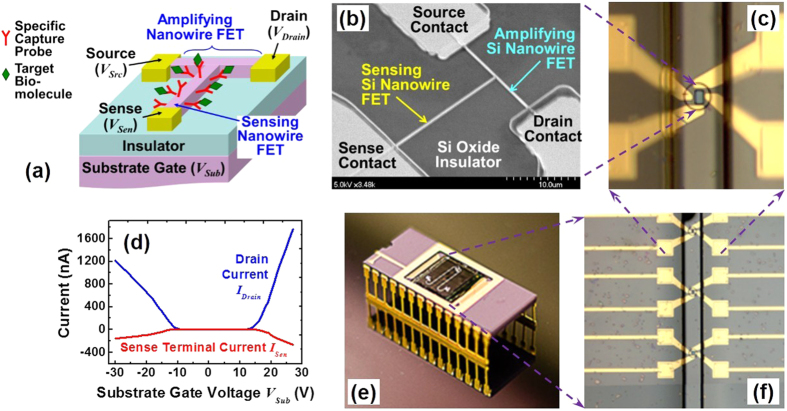
Amplifying T-nanowire FET sensor and Semiconductor Electronic Label-Free Assay (SELFA). (**a)** Structure of the label-free amplifying T-nwFET biosensor with integrated sensing and amplifying nwFET. (**b)** SEM image of a T-nwFET prototype fabricated on an SOI substrate. The Si nanowire width is 100 nm and the source, drain and sense contacts are made of Au-on-Ti. (**c)** Optical micrograph of an SU-8 microfluidic well exposing the T-nwFET’s sensing nanowire surface. (**d)** Measured drain and sense currents versus varying substrate gate voltage of a dry T-nwFET in air. (**e)** Photograph of a ceramic dual inline package embedding an SOI chip integrated with 24 T-nwFET sensors along two microfluidic channels, constituting the optics-free and enzyme-free SELFA. (**f)** Optical micrograph of an array of T-nwFET sensors along one integrated microfluidic channel.

The amplifying FET sensor in SELFA has a compact T-shape nanowire structure, integrating therein two nanowire FETs – one for sensing and the other for amplification (Fig. 1a). We denote the compound device as a T-nwFET sensor. Resting on an insulator, the T-nwFET sensor embraces the genuine advantage behind the “large surface area-to-volume ratio” preception^22^ via the sensing nwFET, and offers electrical signal amplification in extreme proximity via the connecting amplifying nwFET (see Supplementary Data Fig. 2 for amplification mechanism^20^,^21^). We fabricated our T-nwFET sensors on silicon-on-insulator (SOI) substrates using a combination of electron-beam and optical lithography as well as other standard semiconductor fabrication processes (for more details see the ‘Device fabrication’ subsection of the Methods section). The widths of Si nanowires of the fabricated T-nwFET sensors were around 100 nm, as confirmed using scanning electron microscopy (SEM), and shown in Fig. 1b. We further passivated the T-nwFET sensor substrate surface with a 1.5-μm thick SU-8 polymer layer to prevent electrical shorts. The SU-8 in the area immediately above and nearby the sensing Si nanowire was removed via a photolithography step, with the opening becoming part of a microfluidic well for interfacing with reagents and analyte solutions (Fig. 1c,f). Prior to testing in buffer solutions, we verified dry T-nwFETs’ intrinsic amplification function in air (see ‘Electrical measurement’ section of the Methods for more details). In Fig. 1d, the qualitative analysis showed that the T-nwFETs’ drain current *I*_*Drain*_ (blue line) did change more rapidly than the corresponding sense current *I*_*Sen*_ change (red line) upon varying the substrate gate voltage, *V*_*Sub*_. We should clarify though the *I*_*Sen*_ changes due to biosensing as discussed below were not induced by any variation in *V*_*Sub*_.

For subsequent measurement portability, we packaged the SOI chip containing 24 T-nwFET sensors inside a ceramic dual inline package (DIP) via wire bonding (Fig. 1e), allowing concurrent quantitation of multiple analytes as a panel. We selected human inflammatory cytokine interleukin-1 beta (IL-1β) as a representative cell signaling secretome biomarker for device evaluation purposes without losing generality. IL-1β is a well-studied member of the interleukin family of cytokines which has an intermediate molecular weight of 17 kDa and isoelectric point (pI) of around 6.2^23^. More importantly, IL-1β plays an important role in pathological fibrosis in which it is secreted by macrophages receiving toxicants such as engineered nanomaterials (ENMs), and is central to the regulation of inflammatory responses including acute pulmonary inflammation^24^,^25^. We immobilized the exposed sensing Si nanowire surface of the T-nwFETs in SELFA package with anti-human IL-1β monoclonal antibody (mAb) capture probes. We also passivated the remaining surface with amine-terminated polyethylene glycol (PEG) to suppress random biofouling or non-specific binding (for more details see ‘Surface modification’ subsection of the Methods section). The resultant optics-free packaged SELFA is readily interfaced with portable readout electronics for *in silico* data processing, storage, and transmission, including the on-site measurement and data recording in Biosafety Level 2 laboratories.

**Figure 2:**
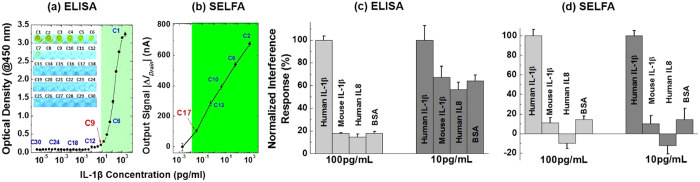
Evaluation of lower limit of detection (LLOD) and cross-reactivity with interferents. Steady-state calibration curve for (**a)** ELISA, and (**b)** SELFA. Measured interferent responses for (**c)** ELISA, and (**d)** SELFA normalized to that of human IL-1β at the same concentration for two interferent concentrations – 10 and 100 pg/mL.

We first examined the LLOD of SELFA versus that of ELISA. To reduce the counter-ion screening effect in FET-based biosensing for SELFA, we prepared 0.01 × phosphate buffered saline (PBS) solutions with different human IL-1β concentrations through serial dilution from a spiked starting concentration of 2,000 pg/mL (*C*_1_) to 3.7 × 10^−6^ pg/mL (*C*_30_). We performed quantitative ELISA according to the kit manufacturer’s instructions (for more details see ‘ELISA procedures’ subsection of the Methods section). After sufficient binding and secondary conjugation, we measured the optical density (OD) values at 450 nm using a microplate reader for the aforesaid human IL-1β concentration series as shown in Fig. 2a. We found the ELISA kit’s LLOD to be ~5 pg/mL (between *C*_9_ and *C*_10_), a value which is close to the manufacturer’s specification and corroborated by the respective bright field images shown in the inset of Fig. 2a. The dynamic range (DR) of ELISA turned out to be about 2 orders of magnitude.

The same human IL-1β samples were used to test the performance of our T-nwFET in SELFA. In brief, we first introduced the pure 0.01 × PBS solution to the T-nwFET sensors and measured the respective *I*_*Drain*_ as blank-signal levels (*I*_*Drain_bl*_). This was followed by a 10-minute incubation step, which is substantially shorter than that required in typical ELISA, to allow sufficient IL-1β binding with the mAb probes pre-immobilized onto the sensor’s nanowire surface. With the solutions in place, we measured the steady-state drain current *I*_*Drain_ss*_ (and steady-state sense current *I*_*Sen_ss*_) at few different *V*_*Sub*_ while holding the source, drain and sense terminal voltages (*V*_*Src*_, *V*_*Drain*_, and *V*_*Sen*_) constant. Finally, we computed the differences between *I*_*Drain_ss*_ and *I*_*Drain_bl*_ as |Δ*I*_*Drain*_| (equivalent to |Δ*G*_*Drain*_| with constant *V*_*Drain*_)^26^, and plotted them against the corresponding human IL-1β concentrations (Fig. 2b). We extracted the LLOD of SELFA’s T-nwFET to be ~10 fg/mL, which we define here as the concentration that corresponds to the |Δ*I*_*Drain*_| value equals to 3 times of the *I*_*Drain_bl*_ noise^27^. In addition, we observed the DR of SELFA to be around 4–5 orders of magnitude, which could be useful for quantitating specimens with wider range of biomolecular abundance.

The SELFA paradigm improved upon the LLOD of ELISA by almost 3 orders of magnitude. This was made possible because the attainable output-signal levels in SELFA’s T-nwFET sensors were substantially higher than those in generic nwFET sensors. The typical T-nwFET’s |Δ*I*_*Drain*_| was ~170 nA at ~100 fg/mL of IL-1β (the corresponding drain conductance change |Δ*G*_*Drain*_| is ~85 nS) while that of a typical generic nwFET at ~100 fg/mL of IL-6, another cytokine, was ~90 pA (with |Δ*G*_*Drain*_| of ~450 pS)^28^. Incidentally, the LLOD of that generic nwFET was ~100 fg/mL as well^28^ (see also Supplementary Data Fig. 3 for our own generic nwFET data).

Besides LLOD and DR, we evaluated both SELFA and ELISA for any cross reactivity with interferents that might lead to false-positive or inaccurate results. We selected three representative and biologically relevant interfering molecules with respect to the human IL-1β target. The first was mouse IL-1β due to its structural similarity to the target and thus potentially high cross reactivity with the anti-human IL-1β mAb capture probes. The second was human IL-8 in the same interleukin family which molecular weight of 11.1 kDa is similar to human IL-1β. The third molecule was bovine serum albumin (BSA) which is an abundant protein with low cross reactivity.

We prepared 0.01 × PBS solutions of each of the three interferents at two concentrations (*i.e.*, 10 and 100 pg/mL), as well as the human IL-1β calibration standards of the same concentrations. We followed the aforementioned ELISA and SELFA procedures and collected the cross reactivity results as shown in Fig. 2c,d. For ease of comparisons between ELISA and SELFA, we normalized all the measured interferent responses with respect to that of human IL-1β of identical concentrations. Also, it is worth noting that the SELFA responses should be positive for mouse IL-1β and BSA since the *p*I values of both molecules (4.56 and 4.7, respectively^29^, signaling-gateway.org) are below the pH value of 0.01 × PBS solution (~7.4); conversely, the SELFA response should be negative for human IL-8 due to its *p*I value (9.1, signaling-gateway.org).

With 100 pg/mL of interferents (*i.e.*, mouse IL-1β, human IL-8, or BSA), we observed almost equivalent selectivity between ELISA and SELFA, with averaged responses in absolute amplitude being 16.74% and 11.78%, respectively. At interferent concentration of 10 pg/mL, approaching the ELISA’s LLOD, ELISA generated an average response of 62.50% and thus became incapable of differentiating between interferent and target molecules. On the contrary, SELFA still maintained its fidelity yielding an average response of 12.15%.

In summary, SELFA is able to offer a substantially lower analytical LLOD compared to ELISA. This is made possible by the reagent-less and local signal transduction and amplification mechanisms harnessed by the T-nwFET sensors in SELFA. In addition, the label-free SELFA exhibits comparable analytical selectivity versus the labeling-based ELISA, validating the reliability of our SELFA platform.

### SELFA implementation for predictive toxicology – A case study in acute lung inflammatory toxicity

We and others have previously developed a predictive toxicological approach for ENMs such as redox-active metal oxides and long aspect ratio (LAR) materials^2^,^30^ based on the assessment of pro-inflammatory and pro-fibrogenic cellular responses. In particular, we examined their toxicities via quantification of secretome biomarkers (*e.g.* IL-1β, other cytokines, and growth factors) that is routinely performed using relatively expensive, time-consuming, and labor-intensive ELISA assays in 96-well plates^31^,^32^,^33^,^34^,^35^. To demonstrate the utility of SELFA secretomics in predictive toxicology, we performed comparative analyses between SELFA and ELISA in quantitating IL-1β released in cultured macrophage receiving some new as well as previously examined ENMs. SELFA quantification of IL-1β release generates *in vitro* hazard ranking of the examined ENMs that directly indicates their degree of lysosomal damage in cells and acute inflammation in animal lungs. The presented SELFA platform and procedure development, side-by-side comparison, and the confirmatory cellular and animal study serve as the first demonstration of its application in a real-life toxicological scenario.

**Figure 3:**
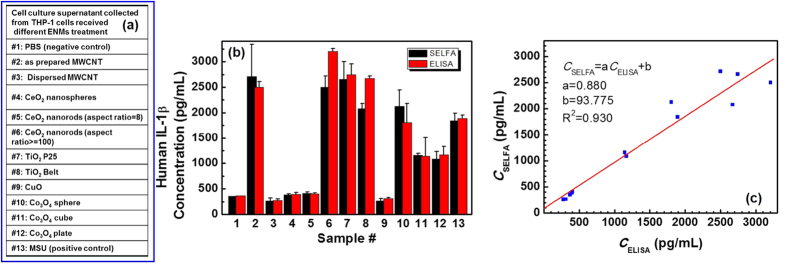
*In vitro* biomolecular assays for *in vivo* toxicology prediction. (**a)** Various metal oxide and long aspect ratio ENMs were used to treat human macrophage cells (THP-1). PBS and MSU solutions served as the negative and positive control, respectively. (**b)** SELFA and ELISA quantitated concentration of human IL-1β in supernatants collected from THP-1 cells which received indicated treatment at 40 μg/mL for 24 hours. (**c)** Correlation between SELFA and ELISA quantitation. Quantitated concentrations of 13 samples (controls included) were linearly fitted, yielding a slope of 0.880 and an intercept of 93.8 pg/mL. *R*^2^ = 0.930.

First, we utilized both SELFA and ELISA to quantitate human IL-1β in supernatants from cultured human macrophage cells (THP-1) receiving a library of ENMs (see the ‘Biological sample preparation’ subsection of the Methods section for more details). We obtained 11 well-characterized ENMs from the UC CEIN compositional and combinatorial material library^30^ for this validation study as tabulated in Fig. 3. In this set of ENMs, the toxicity of #2–#9 were previously assessed while #10–#12 were newly synthesized with unknown toxicity. The morphology of these ENMs was shown in TEM images in Supplementary Data Fig. 4. We also included PBS solution as a negative control as well as the supernatant collected from monosodium urate crystals (MSU) treated THP-1 cell culture as a positive control.

For reliable quantitation of human IL-1β in these biological samples, we developed a novel strategy to calibrate SELFA on a per-sensor basis (for more details see the ‘Sensor calibration’ subsection of the Methods section). In essence, we introduced to each T-nwFET sensor standard solutions whose concentration was below and/or above the anticipated concentration in all supernatant samples to establish the reference calibration points. We were then able to extract the human IL-1β concentrations with confidence by interpolation from the calibration points. The reliable extraction was made possible by correcting for the effects of sensor-to-sensor variations inherited from fabrication and/or probe immobilization (see Supplementary Data Fig. 5 for representative calibration and extraction results). It should be noted that this per-sensor calibration strategy can be simplified to calibrate only a few sensors within a batch so long as the variation among individual sensors is generally minimal.

Next, we deployed and validated the holistic SELFA immunoassay including the hardware, assay procedures, and calibration strategy through determination of human IL-1β concentration in the aforementioned supernatant and control samples. We diluted the supernatants by 250 times in 0.01 × PBS solution such that their concentrations were within the DR of T-nwFET sensors. We measured each sample using at least 2 different sensors, and under multiple voltage biases to further minimize run-to-run variation like running ELISA in duplicates. The SELFA-quantitated concentrations along with the ELISA values for the supernatants under test (Samples #2–12) and controls (Samples #1 and #13) are shown in Fig. 3b, demonstrating good agreement between the two types of assays. For the purpose of method comparison, we further plotted the SELFA-quantitated concentrations against those obtained by ELISA as shown in Fig. 3c. SELFA demonstrates a linear correlation with ELISA with a *R*^2^ > 0.93. From these THP-1 data, we found that ENMs #2, #6, #7, #8 and #10, resulted in a significant increase in IL-1β production compared to PBS treated cells (#1), while ENMs #11–12 led to intermediary elevation. Sample #13, which is MSU positive control, yielded an expected increase in the level of IL-1β.

**Figure 4:**
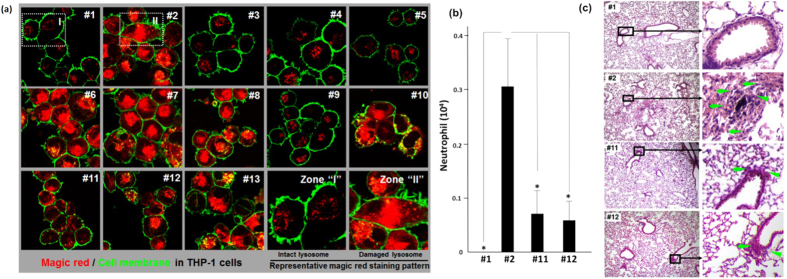
*In vitro* and *in vivo* experimentation confirming SELFA prediction of lung inflammation potential. (**a)** Visualization of cathepsin B localization in THP-1 cells after ENMs treatment. After ENMs treatment, cells were stained with Magic Red (for identifying lysosomal damage and cathepsin B release) and wheat germ agglutinin-Alexa Fluor 488 (for identifying cell membrane), followed by visualization under a confocal 1P/FCS inverted microscope. (**b)** Anesthetized C57BL/6 mice were exposed to the selected ENMs once via oropharyngeal aspiration and were euthanized after 48 hours. The BAL fluids were collected to determine the neutrophil count. (**c)** Lung tissues were harvested, fixed, and stained with hematoxylin/eosin (H&E). While a small increase in the level of pulmonary inflammation (marked by arrows) was observed for samples #11 and #12 compared to the control (#1), a dramatic acute pulmonary inflammation effect was apparent from #2.

To further validate the toxicity ranking generated by SELFA, we carried out confocal microscopy study in THP-1 cells, animal experiments, and histology assessments. It has been established that IL-1β production in macrophages requires assembly of the NLRP3 inflammasome, which activation is dependent on lysosome damage and the lysosomal enzyme cathepsin B release following the endocytosis of these ENMs^36^,^37^,^38^. Therefore, toward confirming the foregoing IL-1β ranking, we used confocal fluorescence microscopy to discern lysosomal damage by tracking the containment of cathepsin B that could be detected by Magic Red probes in THP-1 cells (see the ‘Confocal microscopy assay’ subsection of the Methods section for more details). As shown in Fig. 4a, PBS-treated cells (Sample #1) revealed a punctate distribution of red fluorescence in the intact lysosome, which is in marked contrast to the diffuse staining pattern observed in MSU-treated cells (positive control Sample #13). Consistent with the IL-1β data (Fig. 3) which identified ENMs (#2, #6, #7, #8 and #10) that triggered strong cytokine release, the Magic Red staining revealed the same set of ENMs that induced obvious cathepsin B release and potent lysosome damage, while ENMs #11–12 incurred intermediary lysosomal damage with a corresponding intermediary level of IL-1β production. The remaining ENMs did not provoke any observable lysosomal damage, as expected from the foregoing SELFA IL-1β cytokine analysis.

Although we have demonstrated above an excellent correlation between the IL-1β data and confocal visualization, we have yet to show the former’s indication of pulmonary toxicity in animal lungs. In other words, we need to examine how well the SELFA-based cytokine ranking *in vitro* predicts the degree of acute lung inflammation *in vivo*. Due to logistic constraints (*e.g.* number of animals), we decided to validate the induced lung damage for selected ENMs, *i.e.* #2 (which was assessed before and with predicted high toxicity by SELFA), #11 and #12 (which were new ENMs with predicted intermediary toxicity) in an acute toxicity experiment *in vivo*. We compared the acute pro-inflammatory effects of introducing 5 μg of both ENM to the animal lungs. We chose this dose based on our experiences and its relevance to occupational exposure level estimated by dosimetry^39^. After 48 hours post oropharyngeal aspiration, we collected the bronchoalveolar lavage (BAL) fluid and determined the neutrophil count which is an acute response to inflammatory lung injury (see the ‘Animal experiment’ subsection of the Methods section for more details). We found that the neutrophil count in BAL fluid from animal treated with ENM #2 (as-prepared multiwall carbon nanotube) was significantly higher than that with #1 (PBS negative control) (Fig. 4b), which is in agreement with our previous results^31^. On the other hand, the ENMs #11 and #12 (Co_3_O_4_ nanocube and plate, respectively) triggered a weaker response compared to #2 yet the corresponding neutrophil count was still higher than that for #1. We further confirmed the increased potency of ENM #2 via histology of the corresponding lung tissue (Fig. 4c), which showed the typical focal areas of acute inflammation surrounding small airways in the lung (marked by arrows), in contrast to the less severe inflammation in the animal receiving #11 and #12. At the same time, we observed no acute inflammation with the negative control (#1) as expected. Concisely, Fig. 4 constitutes the integrated set of animal validation data that confirms the same degree of ENMs *in vivo* pulmonary toxicity as predicted by the *in vitro* toxicological cytokine ranking generated by the novel SELFA platform.

## Discussion

Although a FET for detecting immunoreaction has existed for over 3 decades, a practical FET immunoassay with validated operation and calibration procedures that can quantitate biological specimens in the scenario of interest remains lacking. We have proposed and demonstrated in this work a label-free, high-sensitivity biomolecular assay platform, SELFA, based on our amplifying T-nwFET biosensor. We examined its sensitivity and selectivity to a cytokine of interest to predictive toxicology (human IL-1β), and compared the results with an ELISA assay. The results revealed that SELFA has an LLOD ~10 fg/mL, or almost 3 orders of magnitude lower than the conventional ELISA approach, and a wider dynamic range spanning across 4–5 orders of magnitude. Moreover, in the presence of various interfering species, SELFA exhibits a high selectivity to human IL-1β comparable to ELISA. We further evaluated SELFA secretomics in predicting the inflammatory toxicity of various ENMs *in vitro*. Using a novel per-sensor calibration strategy, SELFA is able to quantitate the secreted human IL-1β concentrations in cultured human macrophages receiving different ENMs. The quantitation results agreed well with that of ELISA and corroborated the confocal visualizations of IL-1β related lysosomal damage. Most importantly, the SELFA-generated *in vitro* toxicity ranking was further substantiated by an acute lung inflammation study on animals instilled with selected ENMs. In sum, the holistic SELFA platform and procedures is able to quantify key biomarkers in real biospecimens and readily deployed for predictive toxicology studies.

## Methods

### Device fabrication

We fabricated T-nwFET devices on (100)-oriented *p*-type silicon-on-insulator (SOI) wafers (SOITEC, USA). We patterned the silicon nanowires via e-beam lithography, chrome layer evaporation, photoresist liftoff, and subsequent reactive ion etching (RIE) of the top SOI layer not covered by chrome. Then we formed the contact electrodes via photoresist lift-off of an evaporated gold-on-titanium layer followed by rapid thermal annealing at 450 °C for 60 seconds. Next, we spin-coated a 1.5 μm SU-8 layer for surface electrical insulation with openings above and close to the sensing nanowire developed away. The SU-8 layer prevents shorting of metal contacts in the presence of buffer solutions and its hydrophobic surface facilitates biomolecular diffusion to above the sensing nanowire. After immobilizing the sensing surface with antibody capture probes, we packaged the sensor chip into a DIP carrier for portable electrical measurements.

### Electrical measurements

We perform all electrical measurements using the Keithley 4200 Semiconductor Characterization System. We applied *V*_*Drain*_ = 2 V and *V*_*Sen*_ = 0.8 V to the drain and sense terminals, respectively, and grounded the source terminal. We swept the substrate bias *V*_*sub*_ from 15 V to 30 V between which the T-nwFET device operates in the linear regime. Prior to the introduction of biological samples, we measured the baseline drain current *I*_*Drain*_. In all sensing experiments using SELFA, we took steady-state measurements in 0.01 × PBS solutions to ensure a sufficiently long Debye length.

### Surface modification

We functionalized the sensor surface with a 2-step self-assembled monolayer (SAM) layer to allow aldehyde-terminated groups to covalently bond to the antibodies of interest. We first treated the native oxide covering the T-nwFET sensing surface with 2% (v/v) 3-aminopropyltriethoxysilane (APTES) solution (Sigma-Aldrich). Next, we introduced the homobifunctional crosslinker glutaraldehyde (2% v/v) (Sigma-Aldrich) to add an aldehyde moiety to the surface. Subsequently, we coated T-nwFET surface with 10 μg/mL monoclonal anti-human IL-1β antibody in order to covalently attach antibodies to the surface through their amine moiety. Finally, we introduced 1 mg/mL amine-polyethylene glycol (PEG) to block unreacted aldehyde groups and suppress random fouling of the sensor.

### ELISA procedures

We measured the IL-1β concentrations in spiked PBS solutions and cultured THP-1 supernatants using the OptEIA ELISA kit (BD Biosciences, CA) according to the manufacturer’s instructions. In brief, we first coated the 96-well plate with monoclonal anti-human IL-1β antibody. After blocking the surface with PBS supplemented with 10% fetal bovine serum to suppress random fouling, we added 50 μL of calibration and biological samples into the 96-well plate for overnight incubation at 4 °C. Next, we conjugated the captured cytokines with secondary polyclonal anti-human IL-1β cross-linked to horseradish peroxidase. In order to minimize non-specific bindings, we performed multiple washing steps per the instructions including 3 washes after sample incubation, 3 washes after secondary antibody conjugation, and 7 washes before measurement. We measured the absorbance at 450 nm using a plate reader (SpectroMax M5e, Molecular Devices Corp., Sunnyvale, CA, USA).

### Biological sample preparation

We obtained the THP-1 cells from ATCC (Manassas, VA). We cultured the THP-1 cells in RPMI 1640 medium supplemented with 10% fetal bovine serum at 5% CO_2_ and 37 °C. We seeded aliquots of 5 × 10^4^ THP-1 cells in 0.1 mL complete medium with 1 μg/mL phorbol 12-myristate 13-acetate (PMA) overnight in 96-well plates (Corning, NY, USA). We primed the cells with 10 ng/mL lipopolysaccharide (LPS) to initiate transcriptional activation of the IL-1β promoter. We prepared fresh ENM suspensions (40 μg/mL) in complete RPMI medium and sonicated them before use. After 24 hours post ENMs introduction, we separated the ENMs from the supernatant via a centrifugation process at 15,000 rpm for 10–15 minutes. Such procedure had been confirmed to be effective through the use of ICP-OES elemental analysis and TEM observation^34^,^35^. After that, we measured the secreted IL-1β concentrations in resultant culture medium using IL-1β ELISA kit and SELFA.

### Sensor calibration

To minimize the impact of sensor-to-sensor variations due to fabrication or surface preparation on the T-nwFET sensing reliability, we proposed and implemented the following per-sensor calibration strategy to quantitate IL-1β concentration in supernatant samples. On each antibody and PEG-modified virgin T-nwFET sensor, we first recorded the baseline *I*_*Drain*_ in blank 0.01 × PBS solution as *I*_*Drain_bl*_. Then we measured the *I*_*Drain*_’s in 3 standard spiked solutions that we introduced sequentially in an ascending order of human IL-1β concentration; these concentrations were below those expected of the supernatant samples to be measured. Next we introduced a supernatant sample and noted the corresponding *I*_*Drain*_. After that, we recorded the *I*_*Drain*_ of the last standard solution which human IL-1β concentration was higher than the expected supernatant concentration. Note that all foregoing *I*_*Drain*_’s were steady-state values and we subtracted *I*_*Drain_bl*_ apiece from them as |Δ*I*_*Drain*_|’s. Finally, we fit the lower and higher concentration |Δ*I*_*Drain*_|’s using a standard Langmuir isotherm based expression and interpolated the supernatant |Δ*I*_*Drain*_| value to extract the corresponding human IL-1β concentration as illustrated in Supplementary Data Fig. 5.

### Confocal microscopy assay

We detected cathepsin B in THP-1 cells post-ENM exposure using the Magic Red-cathepsin B substrate. We first treated the THP-1 cells with 40 μg/mL ENMs in complete RPMI 1640 medium for 8 hours. We then washed these cell samples with PBS solution, stained them with Magic Red (ImmunoChemistry Technologies) for 1 hour, and fixed them in 4% paraformaldehyde for 20 minutes. After two subsequent washes in PBS solution, we stained the cell membrane with Alexa Fluor 488-conjugated wheat germ agglutinin at room temperature for 1 hour. Finally, we visually examined these cells under a confocal microscope (Leica Confocal SP2 1P/FCS) in the UCLA/CNSI Advanced Light Microscopy/Spectroscopy Shared Facility. We obtained high-magnification images with a 100× objective. We used cells treated with PBS solution and 50 μg/mL of MSU as the negative and positive control, respectively.

### Animal experiment

In order to validate the *in vitro* cytokine data, we performed an animal experiment to assess the acute toxicological responses in the animal lung by oropharyngeal aspiration. We purchased eight week old male C57Bl/6 mice from Charles River Laboratories (Hollister, CA). We housed all mice under standard laboratory conditions that were set up according to an approved protocol. All procedures were conducted in accordance with Division of Laboratory Animal Medicine (DLAM) guidelines and with the approval of the Ethical Committee and Animal Research Committee of UCLA (2004-022-41). We exposed the mice to various ENMs via oropharyngeal aspiration as previously described^39^. In brief, we anesthetized the mice by intraperitoneal injection of ketamine (100 mg/kg)/xylazine (10 mg/kg) with a total volume of 100 μL. With each anesthetized mouse held in a vertical position, we instilled 50 μL of a suspension with 5 μg of one ENM in water (equivalent to 0.25 mg/kg) at the back of the tongue to allow aspiration in the lung. Control mice received the same volume of PBS solution (negative control). We sacrificed the mice after 48 hours post-exposure and collected the BAL fluid and lung tissues. We used the BAL fluid to perform total and differential neutrophil cell counts. We stained histological sections with hematoxylin/eosin to reveal the level of pulmonary inflammation.

## Additional Information

**How to cite this article**: Mao, Y. *et al.* Semiconductor Electronic Label-Free Assay for Predictive Toxicology. *Sci. Rep.* **6**, 24982; doi: 10.1038/srep24982 (2016).

## Supplementary Material

Supplementary Information
