# Supplementary material for: Semiconductor Electronic Label-Free Assay for Predictive Toxicology
Source: Sci Rep. 2016 Apr 27;6:24982. doi: 10.1038/srep24982 (PMC4846994; doi:10.1038/srep24982)
Supplement: Supplementary Information [file srep24982-s1.pdf]

Semiconductor Electronic Label-Free Assay for Predictive Toxicology

Yufei Mao [1], Kyeong-Sik Shin [1], Xiang Wang[2], Zhaoxia Ji[3], Huan Meng [2,3*], Chi On Chui [1,3,4,5*]

[1] *Department of Electrical Engineering, University of California, Los Angeles, CA 90095, USA*
[2] *Division of NanoMedicine, Department of Medicine, University of California, Los Angeles, CA 90095, USA*
[3] *Center for Environmental Implications of Nanotechnology, University of California, Los Angeles, CA 90095, USA*
[4] *Department of Bioengineering, University of California, Los Angeles, CA 90095, USA*
[5] *California NanoSystems Institute, University of California, Los Angeles, CA 90095, USA*

[*] Corresponding author. Tel.: +1 310 267 4786; fax: +1 310 825 8282. *E-mail address*: chui@ee.ucla.edu (C. O. Chui); Tel.: +1 310 983 3359; fax: +1 310 206 8107. *E-mail address*: hmeng@mednet.ucla.edu (H. Meng)

Supplementary data (one Supplementary data figure per page) .

Supplementary Figure 1. | Reported nwFET biosensor performance with that of SELFA's T-nwFET.

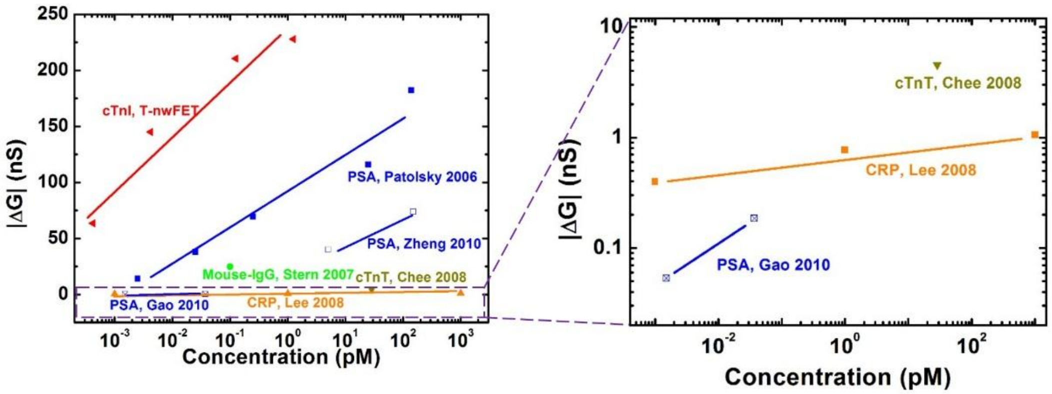

Biosensing performances, especially near the lower limit of detection (LLOD), of silicon nwFET sensors reported in the literature are benchmarked. Also included was the data of a T-nwFET device in red. In spite of the difference in biomarker, a low output signal, i.e. absolute change in output conductance $|\Delta G|$, at LLOD is generally observed for all nwFET sensors. On the other hand, the T-nwFET device (e.g. in quantitating human cardiac troponin I or cTnI) offers a substantially enhanced $|\Delta G|$, making it possible to interface with portable readout electronics.

Supplementary Figure 2. | Equivalent circuit of the T-nwFET device.

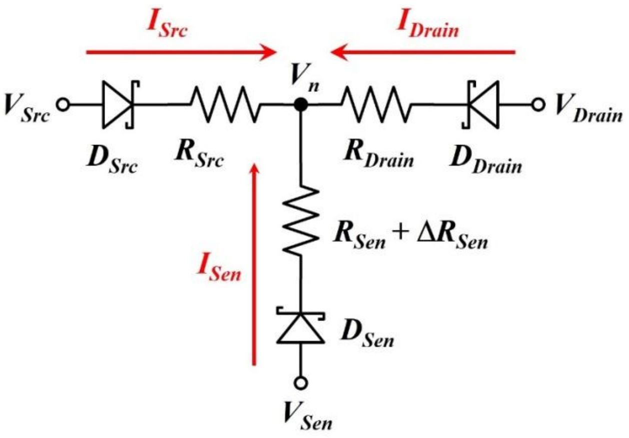

The circuit model above can be used to understand the amplification mechanism[19]. Each nanowire segment from the intersecting node (at a potential of V_n) to the respective electrode can be represented as an ideal Schottky diode in series with the nanowire resistor. Accordingly, we can express the sense current and drain current as:

$$I_{Sen} = I_0 \left[\exp\left(\frac{q\left(\left(V_{Sen} - V_n\right) - I_{Sen}\left(R_{Sen} + \Delta R_{Sen}\right)\right)}{k_B T} \right) - 1 \right]$$

$$I_{Drain} = I_0 \left[\exp\left(\frac{q\left(\left(V_{Drain} - V_n\right) - I_{Drain} R_{Drain}\right)}{k_B T} \right) - 1 \right]$$

$$I_0 = AA^{**}T^2 \exp(-\frac{q\phi_{Bp}}{k_B T})$$

where A, A^{**}, k_B, ϕ_{Bp}, q, R_{sen}, ΔR_{Sen}, R_{Drain} and T are the diode area, effective Richardson constant, Boltzmann constant, Schottky barrier height, electronic charge, sensing nanowire resistance, change in R_{sen} due to sensing, drain nanowire resistance, and absolute temperature, respectively. The sense current I_{sen} and output current I_{Drain} can be related through V_n. Given the exponential behavior of Schottky diodes, any change in V_n

due to ΔR_{Sen} would change the current nonlinearly. A high current amplification ratio (dI_{Drain}/dI_{sen}) can be achieved by proper voltage bias.

Supplementary Figure 3. | Generic non-T-shape nwFET sensing response of human IL-1β in 0.01× PBS buffer.

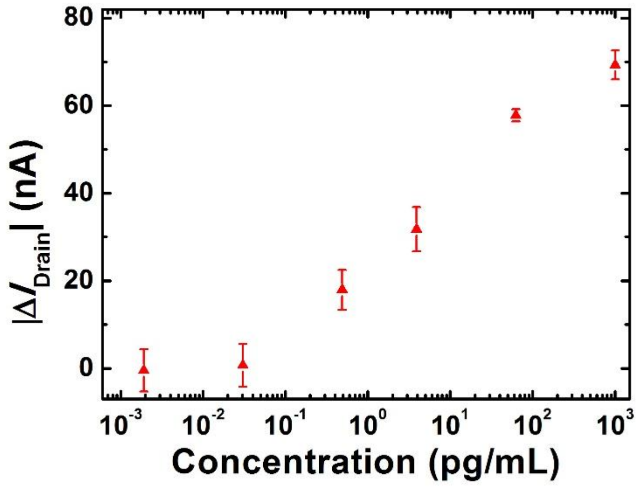

Measurements were taken on T-nwFET sensors with the source terminal floating, and sense terminal grounded. V_{Drain} at 1 V and V_{sub} at 30 V were applied. The current changes $|\Delta I_{Drain}|$ were extracted with reference to I_{Drain_bl} which was measured in blank 0.01× PBS solution without IL-1β.

Supplementary Figure 4. | TEM images of nanoparticles.

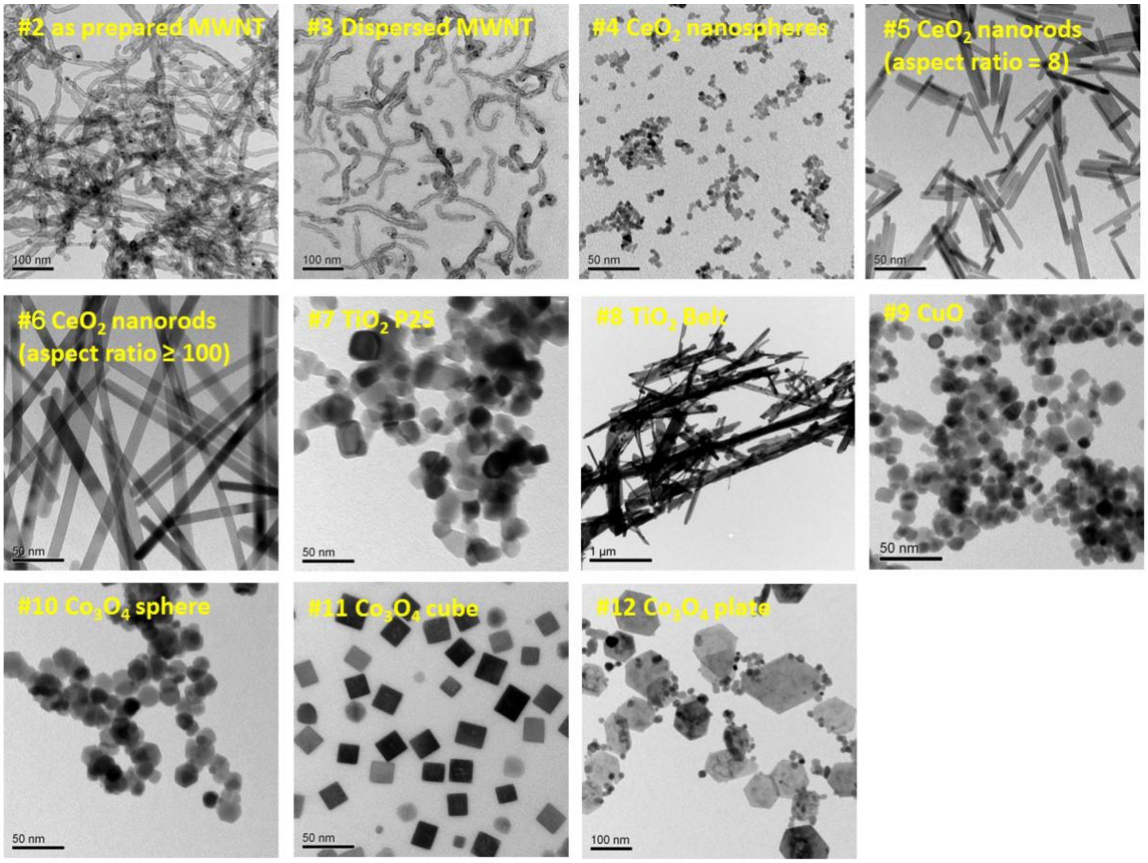

A compositional and combinatorial engineered nanomaterial library was obtained from the UC CEIN nanomaterial core (http://www.cein.ucla.edu/new/p11.php) and used in the current study. The particles were synthesized in-house and fully characterized. The in-house synthesis of materials allowed us to control material quality (e.g. size, shape, purity, dispersion, etc.) as opposed to commercial sources that did not allow exact control of material properties in many instances. A full panel of TEM images of the nanoparticles used in this study is shown.

Supplementary Figure 5. | Representative calibration procedure of SELFA using standard spiked solutions and extraction of human IL-1β concentration in a supernatant sample.

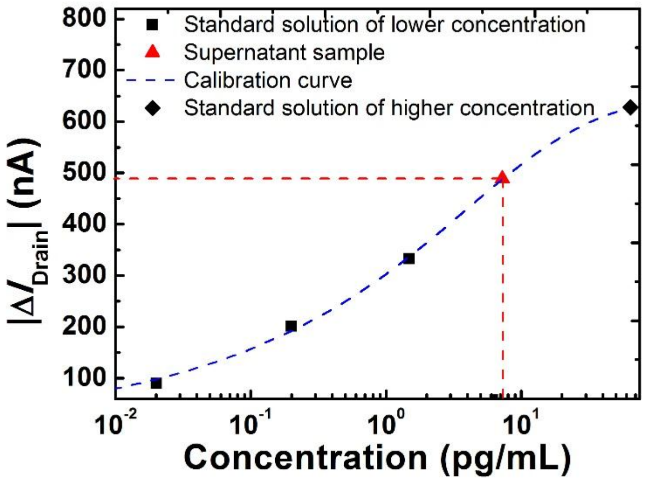

Drain currents in blank PBS solution, three lower standard solutions, the supernatant sample, and one higher standard solution were measured in sequence. The output signals, $|\Delta I_{Drain}|$'s, were computed to interpolate the concentration of the supernatant sample. For optimal sensitivity, the supernatant samples were diluted 250 times in 0.01× PBS so that their IL-1β concentrations fell within the dynamic range of SELFA. The calibration curve was based on the standard Langmuir isotherm model with fitting parameters to account for slight deviation from linearity intrinsic to the nwFET sensor signal transduction.
